# Engineering Precise Interconnected Porosity in β-Tricalcium Phosphate (β-TCP) Matrices by Means of Top–Down Digital Light Processing

**DOI:** 10.3390/biomedicines12040736

**Published:** 2024-03-26

**Authors:** Thomas Wojcik, Feng Chai, Vincent Hornez, Gwenael Raoul, Jean-Christophe Hornez

**Affiliations:** 1 Univ. Lille, CHU Lille, INSERM, Department of Oral and Maxillofacial Surgery, U1008—Advanced Drug Delivery Systems, F-59000 Lille, France; gwenael.raoul@chu-lille.fr; 2 Univ. Lille, CHU Lille, INSERM, U1008, F-59000 Lille, France; feng.hildebrand@univ-lille.fr; 3 CRYOCERAM, F-59144 Bry, France; cryoceram@gmail.com; 4 Département Matériaux et Procédés (DMP), Laboratoire de Matériaux Céramiques et de Mathématiques (CERAMATHS), Université Polytechnique Hauts-de-France, F-59600 Maubeuge, France; jean-christophe.hornez@uphf.fr

**Keywords:** tissue engineering, 3D printing, tricalcium phosphate, digital light processing, ceramic scaffold

## Abstract

This study evaluated the biocompatibility and accuracy of 3D-printed β-tricalcium phosphate (β-TCP) pure ceramic scaffolds. A specific shaping process associating a digital light processing (DLP) 3D printer and a heat treatment was developed to produce pure β-TCP scaffolds leaving no polymer binder residue. The β-TCP was characterised using X-ray diffraction, infrared spectroscopy and the detection of pollutants. The open porosity of produced matrices and their resorption were studied by hydrostatic weighing and calcium release measures. The biocompatibility of the printed matrices was evaluated by mean of osteoblast cultures. Finally, macroporous cubic matrices were produced. They were scanned using a micro-Computed Tomography scanner (micro-CT scan) and compared to their numeric models. The results demonstrated that DLP 3D printing with heat treatment produces pure β-TCP matrices with enhanced biocompatibility. They also demonstrated the printing accuracy of our technique, associating top-down DLP with the sintering of green parts. Thus, this production process is promising and will enable us to explore complex phosphocalcic matrices with a special focus on the development of a functional vascular network.

## 1. Introduction

Bone is a living tissue with its own regenerative properties, enabling it to constantly remodel and adapt. However, major traumas, tumours and aging may create circumstances in which a bone graft needs to be used to create appropriate conditions for bone reconstruction or healing and deal with large bone defects. Therefore, by the 2000s, bone grafting had become a common procedure, with over 2.2 million taking place worldwide each year in the late 2000s; in 2005 alone, these procedures are estimated to have cost over USD 2.5 billion [1]. To date, autologous bone grafts (including bone-free flaps) are the gold standard in managing bone defects and represent approximatively 50% of all procedures undertaken. Autologous bone grafting provides the best osteoconduction and osteointegration properties because of the perfect match in the composition and architecture of phosphocalcic matrices but also because they carry osteogenic cells, osteoregulator factors and collagenic scaffolds. Autologous bone grafts, however, also present disadvantages such as morbidity of the donor site and the limited quantity of bone available [2,3,4,5,6]. As an alternative, allografts and xenografts can be used, which represent 25% of all bone graft procedures performed. In both cases, there is no limit on quantity and no donor site morbidity. However, both possess fewer biological properties and can therefore induce immune responses and present a potential risk of infectious transmission [7,8,9,10].

For decades now, alternative materials have therefore been developed and studied by scientists, and these synthetic grafts now represent up to 25% of all materials used in grafts. These materials aim to reproduce the biological and mechanical properties of native bone in unlimited amounts, with no infectious risk and at a reasonable cost. One of the most popular families of material is the calcium phosphate synthetic apatites. These have been demonstrated to be highly biocompatible, osteoinductive and osteoconductive, even if they do not exhibit these properties at the same level as biological apatites [11,12]. Their properties are dependent on their synthesis procedures, purity and crystal size, as well as their macro- and micro-porosity [13]. The management of the processes involved in both synthesis and shaping is therefore essential in controlling the final properties of the produced matrices.

In this work, we focused on β-tricalcium phosphate (β-TCP), which is a resorbable calcium phosphate (CAP) that can be replaced by native bone [14]. However, β-TCP is currently grafted using powders or preformed graft blocks that do not perfectly match the bone defects in question and present uncontrolled porosity that may lead to unpredictable and non-reproducible results, with potential failure of the procedures. According to current research, the development of three-dimensional (3D) printed matrices can be a game-changer in bone reconstructions [15,16,17,18,19,20,21,22]. In fact, rapid prototyping procedures associating computer-assisted design (CAD) or the use of computed tomography (CT) scanners with precise 3D-printing techniques or additive manufacturing may allow for the production of matrices specific to individual patients. By controlling the macro-architecture of matrices to adapt their shapes precisely to the bone defects they are being used to treat and developing specific porosity that might vary within the same piece, we may be able to enhance bone reconstruction results compared to those of conventional shaping processes [23].

A number of approaches have been developed to produce complex ceramic matrices based on existing 3D-printing techniques [24]. One of the most promising techniques is digital light processing (DLP). DLP technology is very similar to stereolithography (SLA), working with the ultraviolet radiation (UV) photopolymerisation process. But the light source in DLP is more conventional than laser, and the light is applied to the entire surface of the slurry in a single pass, making it faster than SLA. Like SLA, once the exposure composed of phosphocalcic powder and light-curable resin has hardened, the build platform progressively prints the next layer of the structure vertically [25]. Two types of DLP printer are possible, each placing the light source in a different position; one places it above the slurry tank (top-down), while the other places it under the tank (bottom-up). But while DLP is promising, it also presents some constraints. Just as with conventional shaping, the slurry needs a sufficient solid loading of β-TCP to allow for good densification of pieces, preventing it from cracking and enabling it to withstand the required mechanical stresses. Furthermore, the DLP process prefers slurries with a low viscosity, allowing them to level themselves during printing [26].

Depending on the composition of the slurry, the curing speed and depth may affect printing accuracy as a result of light scattering and refraction [27]. Moreover, during the printing process, photopolymerisation results in the emission of heat that may deform the printed parts.

**Figure 1:**
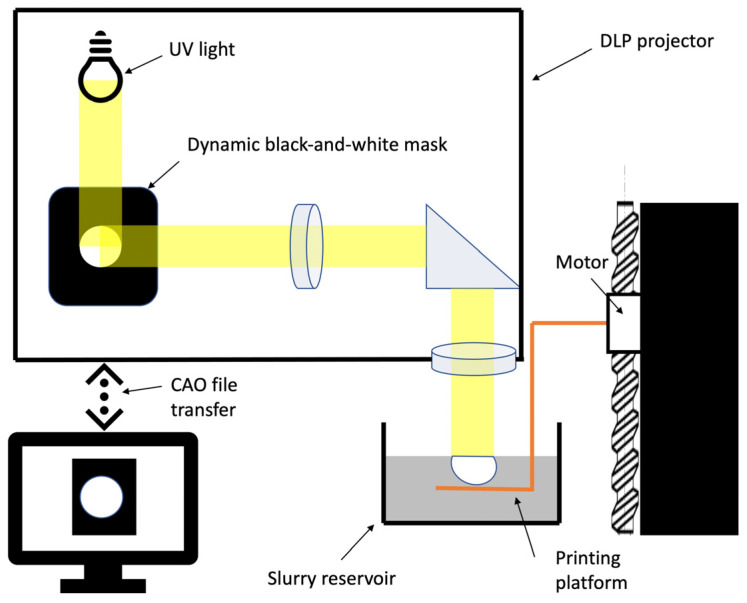
Top–down digital light processing principle.

In this study, we produced β-TCP powder and controlled its composition, thus impacting the biological and mechanical properties of matrices. We decided to develop a top-down DLP printer using a custom slurry to obtain a solid loading identical to the conventional shaping technique (Figure 1).

Following the printing process, the green parts underwent a de-binding heat treatment to remove the resin before a sintering phase, which resulted in the densification of the parts and their final dimensions. The purpose of this preliminary heat treatment is to modulate the resorption of the β-TCP and, thus, its biological and mechanical properties. Due to the shrinkage of the parts, the printing resolution may be improved. However, the shrinkage must be anticipated during their conceptions to obtain final parts with controlled dimensions. Additionally, the use of photopolymerizable resins may have a negative effect on cell growth and proliferation even if the sintering process is supposed to destroy every biological residue. That is why we also controlled the composition of printed matrices and searched residual pollutants to confirm their compatibility with future clinical applications.

The purpose of such shaping techniques including printing steps like DLP is to improve the properties of conventional shaped phosphocalcic matrices. Furthermore, it allows us to explore more complex domains of tissue engineering such as the development of perfusable intra-matricial vascular networks, which are essential for large bone reconstructions. However, preliminary studies are necessary to confirm the accuracy of the printer and the biocompatibility of the printed devices. We therefore focused our research on the following three points: the purity of the produced β-TCP, its biocompatibility and the accuracy of the printing technique.

## 2. Materials and Methods

### 2.1. β-TCP Powder Production

Calcium phosphate powders were prepared using an aqueous precipitation technique with a diammonium phosphate solution ((NH_4_)_2_HPO_4_, Carlo Erba, Val de Reuil, France) and a calcium nitrate solution (Ca(NO_3_)_2_,4H_2_O, Brenntag, Chassieu, France) [28,29]. To synthesise the β-TCP powders, we set the temperature to 30 °C and the pH to 6.4, enabling us to stabilise the TCP phase and avoid the formation of hydroxyapatite. The initial Ca/P molar ratio was 1.5, with an aging time of 20 h for the syntheses. After aging, the solutions were filtered, and the precipitates were dried at 70 °C. Powders were then calcined at 850 °C for 3 h with a speed rate of 5 °C/min. This process of calcination facilitates the transformation of the apatitic calcium phosphate phase into β-TCP, the elimination of residual nitrates and a reduction in the specific surface area, providing a more convenient value for the 3D-printing casting method. Following calcination, the powders were ground for 48 h to break up agglomerates formed during the thermal treatment and reduce the powder to its ultimate particle size. This grinding step was carried out by ball milling using a High-Density Polyethylene (HDPE) milling jar and yttrium-stabilised zirconia grinding media. The specific surface areas of the powders were recorded by the BET method (Micromeritics, Flow Sorb 3).

### 2.2. β-TCP Characterisation

During the synthesis and calcination phases, despite the strictest control of the various parameters, the synthesis of β-TCP may include molecules of hydroxyapatite and/or pyrophosphate calcium in variable quantities. Variations in these proportions have an impact on the biological and physical properties of the materials, especially on their mechanical strength. For characterisation, the calcined powder underwent a new calcination at 1000 °C for 15 h to increase the proportion of crystalline phases. We therefore used X-ray diffraction (XRD) and infrared spectrometry (FT-IR) to study the composition of our powder. However, the powder may contain other phases in quantities too small to be detected by X-rays. In this case, a negative phenolphthalein test indicates that the powder does not contain lime (CaO) traces [30]. Phenolphthalein is a coloured indicator that changes from colourless to pink for a pH greater than 8.2, allowing us to detect traces of CaO.

#### 2.2.1. X-ray Diffraction

To study the potential impact of the DLP printing process on the purity of the β-TCP, three samples of both printed and casted matrices were crushed to obtain powders that were characterised using the same protocol.

Calcium/phosphate ratios (Ca/P ratios) were determined by powder X-ray diffraction analysis (Rigaku Miniflex, Neu-Isenburg, Germany) using the intensity ratio of lines HA (211) and ITCP (0210) according to the method of proportioned additions [31,32]. In this context, the lines of interest are (210) (2θ = 28.971°) or (211) (2θ = 31.772°) for HA and (0210) (2θ = 31.027°) for β-TCP. For proportions of HA less than 30%, however, line (210) is difficult to observe. In this case, we therefore prefer line (211), which remains exploitable for HA levels greater than 0.5%.

#### 2.2.2. Infrared Spectroscopy

The presence of calcium pyrophosphate (CPP) (Ca_2_P_2_O_7_),was controlled by infrared spectroscopy on a Fourier transform spectrometer (Jasco-FT/IR-460 Plus, Lisses, France).

Three samples of both printed and casted matrices were crushed to obtain powders that were characterised using the same protocol.

The infrared spectra were recorded in transmission by a Fourier transform spectrophotometer between 400 and 4000 cm^−1^ (Jasco FT/IR-460plus, Lisses, France) on pellets containing 298 mg of potassium bromide (KBr) and 2 mg of β-TCP powders. These measurements allowed us to determine the presence or absence of CPP in β-TCP by observing the characteristic CPP bands at 720 cm^−1^ and 1200 cm^−1^.

The 757 cm^−1^ and 434 cm^−1^ bands, which indicate the presence of the α form of calcium pyrophosphate, or the 1210 cm^−1^ and 1185 cm^−1^ bands at 723 cm^−1^ and 454 cm^−1^ can also be studied (the latter are characteristic of the β form of calcium pyrophosphate). Thus, the absence of these different bands during the FT-IR analysis indicates undetectable CPP contamination of β-TCP production.

We employed infrared spectroscopy rather than using quantitative techniques such as EDS or WDS because infrared is the most sensitive method for detecting the presence of pyrophosphate in TCP using the absorption band at 721 cm^−1^ that is characteristic of the stretching vibration of the pyro group’s P-O-P bond.

### 2.3. Searching for Pollutants or Trace Elements

To quantify the presence of heavy metals and trace elements, we conducted an analysis using inductively coupled plasma spectrometry. Samples were ionised using a plasma torch and produced ions that were then detected using mass spectroscopy. Two samples were tested. The first was composed of powder sampled after the synthesis process, and the second was a printed tab.

This research was carried out by an external company (FILAB’, Dijon, France), and the elements researched were mercury (Hg), arsenic (As), lead (Pb), cadmium (Cd), antimony (Sb), chromium (Cr), nickel (Ni), barium (Ba) and copper (Cu).

### 2.4. Scaffold Shaping

#### 2.4.1. DLP Rapid Prototyping

##### Computer-Assisted Design Process

Considering our objectives, we decided to design a range of pieces. For the osteoblast culture, we designed non-micro-porous matrices comprising discs of 6 mm in diameter and 2 mm in thickness for introduction in 96-well plates. To study the accuracy of our printing technique, we designed two micro-porous cubes. The first had sides of 5 mm and four square windows on each side of 1 mm by 1 mm. The second of had sides of 10 mm and four square windows, each measuring 2 mm by 2 mm, on each side. All 3D models used in this study were produced using Windows 3D Builder^®^ software (Version 5.2, Mycrosoft, Redmond, WA, USA) for STL files. To consider the shrinkage of samples during the sintering process, they were designed with oversized dimensions. The 3D models were then cut into series of 50-micrometre (µm)-thick layers using Creation Workshop^®^ software (Version1.0.0.75, freeware, Steve Hernandez). The files were then transmitted to the printer control software (Cryoberyl Software Version 1.2, Cryoceram, Bry, France) for production.

##### DLP Printing Technique

**Figure 2:**
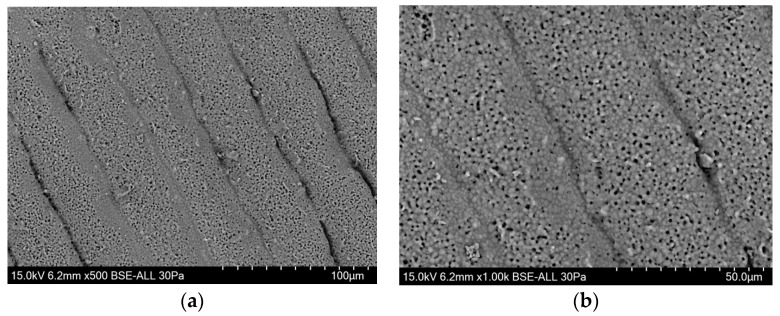
Scan of a printed part using an electron microscope with a resolution of 50 µm. We can see that at 500× (**a**) and 100× (**b**) magnifications, the layers are less than 50 µm thick due to sintering at 1050 °C, but the matrices maintain a high level of open porosity.

We opted for a top-down DLP printing technique and developed a printer accordingly. A DLP projector projects a black-and-white dynamic mask of ultraviolet light onto the slip surface to obtain a polymerisation corresponding to the slice of the object. The used slip combines photopolymerisable resins and a suspension of phosphocalcic powder, as described in Figure 1. The object being created rests on a motorised tray that is progressively immerged in the slip after each layer under the control of a stepper motor mounted on a high-precision linear guide. This controlled descent of the object allows for layers to be progressively added. A mechanical resolution of less than a micron could be obtained in the Z axis, and the DLP projector was able to provide dynamic masks with an XY-axis resolution of close to 40 µm. Our theorical maximal layer/Z resolution was 50 µm before sintering, as illustrated in Figure 2. The maximum printable dimensions are 95 × 50 × 150 mm.

The slip used in this technique is different from those used in conventional casting techniques. The β-TCP powders were incorporated into a photosensitive (l = 350–400 nm) acrylic resin (T4-CaP; CryoBeryl software, Bry, France) to obtain 68% (weight/weight) dry-matter-content slurries that were homogenised at 150 rpm by planetary milling for 30 min (PM100; Retsch, Haan, Germany).

As with conventional slips, the material loading rate has a direct impact on the density and micro-porosity of the final object. Furthermore, to maintain the flatness of the slip surface, no external scraping or levelling devices are used. Therefore, the suspension must have a low viscosity; this gives it a short relaxation time, creating a brief delay between the printing of two successive layers.

Once the layers to be printed had been entered into the printer’s control software, the printer projected a dynamic mask onto the slurry with an exposure time of 6 s per layer. Between each exposure, the sample was immerged in the slip before rising to 50 µm from the surface to polymerise the next layer. A delay of 10 s was required between the printing of each layer to ensure the flatness of the slip surface.

#### 2.4.2. Conventional Shaping Technique

To compare the safety of the printing technique, we shaped conventional matrices. The used β-TCP powder was the same as that employed in both printing and casting techniques. Scaffolds were prepared using a classical template-casting approach.

The β-TCP powder was suspended in a water solution with dispersant (Darvan C) and surfactant (B1001) to obtain 68% (weight/weight) dry-matter-content slurries homogenised at 150 rpm by planetary milling for 30 min (PM100; Retsch, Haan, Germany). The slurry was then cast in a plaster mould designed to obtain discs with diameters of 6 mm. The mould was then placed in heat chamber overnight (for 12 h) at 30 °C.

### 2.5. De-Binding and Sintering

Once they had been shaped, the parts were carefully transferred into a high-temperature furnace (Ecotop 60, ROHDE, Prutting, Germany) to perform both the de-binding and final sintering of the scaffolds in a single step. During the first phase, the temperature was raised 1 °C/min up to a temperature of 600 °C, which was then maintained for 1 h. This first phase of the temperature increase consisted of de-binding the parts and, thus, gradually eliminating the resins and organic compounds. The next phase increased the temperature at a rate of 5 °C/min until the sintering temperature was reached and maintained for 3 h. Finally, the parts were brought back to room temperature according to a temperature descent curve of 5 °C/min.

Sintering temperatures of 1000, 1050 and 1120 °C were chosen so that we could study the open micro-porosity and biological properties of the matrices.

### 2.6. Evaluation of Open Porosity of the Matrices

The porosities of the matrices were established by hydrostatic weighing. This method allows us to calculate the apparent density and open porosity of a sample by measuring its mass under different conditions.

To determinate their dry mass, the samples were dried in a heat chamber for 30 min at 150 °C. They were then placed in a vacuum chamber to achieve a rough vacuum (<1 kPa). These conditions were maintained for a minimum of 30 min before breaking the vacuum and weighing the samples.

The wet mass of the samples was then measured once they had been transferred to a saturator on a support. A vacuum (<1 kPa) was applied for 1 h. When the discs were immersed in distilled water, the vacuum was broken. Once the samples had been completely immersed, the platform was shaken to eliminate the microbubbles coming from the degassing of the water, and they were kept immersed for at least 1 h. The vacuum was put back in place and then broken every 20 min for three cycles, leaving the samples immerged before they were weighed. Finally, the different dry and wet mass measurements were compared in order to determine the porosity of the samples.

Three types of matrices, each with six samples, were produced so that we could study the impact of the sintering temperature at 1050 and 1120 °C and printing thicknesses of 50 and 100 µm (group 1: 1050 °C, 50 µm; group 2: 1050 °C, 100 µm; group 3: 1120 °C, 50 µm).

### 2.7. Evaluation of DLP Printing Accuracy

The sintering temperature plays a major role in the shrinkage of the produced parts, depending on their composition and production parameters. Preliminary tests were carried out to determine the shrinkage induced in our material at different temperatures. Once these data had been collected, we were able to determine that the printed parts needed to be oversized by 21% at 1050 °C to obtain the desired dimensions after sintering. In this study, to confirm the accuracy of our shaping process, we produced cubes with four windows on each side. A landmark was placed on the top of the cube to identify the X/Y/Z axes. We used the same slip as during our different printing tests.

**Figure 3:**
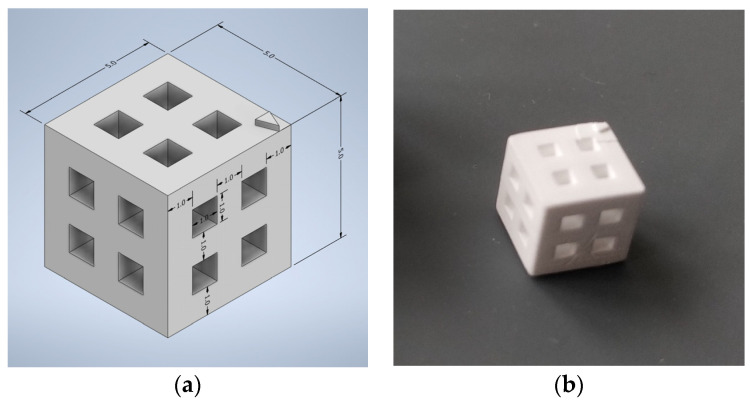
Virtual model of cube before oversizing process (**a**) and result after sintering (**b**).

In the first test, two cubes with sides of 5 mm, each with four windows of 1 mm by 1 mm, were printed and sintered at 1050 °C for measurement (Figure 3).

We performed a second test using two different sets of cube dimensions to confirm our results and the potential impact of the dimensions on the accuracy of the technique. The first cube was identical to those we produced for the first test. The second cube was designed with 1 cm edges, each face having four 2 mm by 2 mm windows. Just as in the first test, the sintering temperature was set at 1050 °C.

We assessed the accuracy of printing using a micro-computed tomography scanner (micro-CT) (Bruker Skyscan 1172, Billerica, MA, USA) and measured it using Geomagic Control X^®^ (Version 2023.1.0, 3dsystem, Rock Hill, SC, USA). The dimensions of the edges were measured using the average dimensions between all the points of a face and their orthogonal antagonists on the opposite face.

### 2.8. Measure for Releasing Calcium Ions

To study the ionic release of our material, we observed the conductimetry variation of distilled water, in which we immersed printed pellets that were 11.5 mm in diameter and 2.1 mm in thickness with a mass of 0.55 g. Pellets were printed according to our technique and then sintered. We studied the effects of three temperatures, namely 1000 °C, 1050 °C and 1100 °C, and sintered two pellets for each temperature. Finally, for each temperature, one pellet was washed for 5 min and the other for 1 h before being sterilized at 170 °C for 1 h. We developed a specific device to test eight samples in parallel, leaving the samples in pure water for 6 days. To convert these conductimetry results into a Ca^2+^ assay, we used an AQUION ICS900^®^ (Thermo Fischer Scientific, MA, USA) ion-exchange chromatography system.

In summary, we studied ionic release according to the following parameters:

- β-TCP with 5 min wash and 1000 °C sintering;
- β-TCP with 1 h wash and 1000 °C sintering;
- β-TCP with 5 min wash and 1050 °C sintering;
- β-TCP with 1 h wash and 1050 °C sintering;
- β-TCP with 5 min wash and 1100 °C sintering;
- β-TCP with 1 h wash and 1100 °C sintering.

### 2.9. Cell Proliferation

#### 2.9.1. Evaluation of Impacts of Sintering Temperatures and Shaping Techniques

To evaluate the biocompatibility of our technique, we decided to compare it to the classic casting technique. Thus, we printed β-TCP pellets and cast another series using β-TCP belonging to the same production. These pellets had a circular shape suitable for culture in 24-well plates. Two sintering temperatures were selected, namely 1000 °C and 1100 °C. The objective of these two temperatures was to study the impact of micro-porosity and matrix degradation on cell culture. Prior to culturing, the pellets were rinsed with pure water for 5 min before being sterilised at 170 °C for 1 h.

In addition, two durations of cultures were studied, with cell counts carried out at 3 and 6 days.

We formed the following groups, each containing six samples:

- β-TCP cast and sintered at 1000 °C;
- β-TCP printed and sintered at 1000 °C;
- β-TCP cast and sintered at 1100 °C;
- β-TCP printed and sintered at 1100 °C;
- MC3T3-E1 on well bottom (control group).

Cells selected for this test were MCT3T3-E1, an immortalised cell line derived from mouse calvaria osteoblast precursors.

Each well was inoculated with 8000 cells in suspension, and 900 µL of specific culture medium was added to each well. Following this, the culture plate was incubated in an enclosure (CB 150/APT line/Binder, LabExchange, Paris, France) with the following parameters: temperature at 37 °C, atmosphere at 5% CO_2_ and 100% water saturation.

The vitality of the cells was studied using a redox indicator, namely Alamar Blue. The culture media were cleaned from each well, then replaced with a culture medium containing 10% fluorescent Alamar^®^ Blue dye (Interchim, Montlucon, France), before being re-incubated for 2 h. Alamar blue, or resurazine, is a blue reagent which, when reduced, transforms into resorufin, which has a highly fluorescent-pink colour. It is therefore used as a marker of cell viability by highlighting the presence of redox activity linked to the number of cells present and their mitochondrial activity.

The solutions were then transferred to 96-well plates (Nunc, Thermo Fischer Scientific, MA, USA), and the fluorescence absorption was measured by a fluorometer (TwinkleTM LB 970, Berthold Technologies, Bad Wilbad, Germany) at 530 nm for excitation and 590 nm for emission. This made it possible to assess the mitochondrial activity and, in turn, the vitality of the different groups.

The vitality rate of the cells in the different groups was calculated by realising the ratio between their absorbance and that of the control group.

#### 2.9.2. Impact of Rinsing the Matrices

In order to improve our results, we modified our protocol to evaluate the impact of subjecting the matrices to a longer period of rinsing. Longer rinsing is supposed to eliminate the most unstable phases of the matrices and thus limit the release of ions during cell culturing, which can be detrimental to their survival. Similarly, daily renewal of the culture medium from the 24th hour of culturing was studied on cells cultured on a dish bottom and on matrices rinsed for 1 h.

We therefore cultured MC3T3-E1 cells on β-TCP matrices shaped by our printing technique and sintered at 1050 °C. The cell culture and vitality evaluation protocols were otherwise identical to the previous biocompatibility test.

We formed the following groups, each containing six samples:

- β-TCP with 5 min wash;
- β-TCP with 1 h wash;
- β-TCP with 1 h wash and daily medium change;
- MC3T3-E1 on well bottom with daily medium change;
- MC3T3-E1 on well bottom (control group).

## 3. Results

### 3.1. β-TCP Characterisation

#### 3.1.1. X-ray Diffraction

During our analysis of both casted and printed matrices, we observed the same elements and similar graphs. Study of the X-ray diffraction graph shows the presence of a large peak of β-TCP, while those of hydroxyapatite and pyrophosphate are absent (using a 2 theta angle of 31.773°, XRD is the most sensitive method to detect the presence of traces of HA in TCP).

#### 3.1.2. Infrared Spectroscopy

Furthermore, infrared spectroscopy graph analysis matches our X-ray analysis, and we observed the absence of characteristic pyrophosphate bands (using the absorption band at 721 cm^−1^ characteristic of the stretching vibration of the pyro group’s P-O-P bond, infrared is the most sensitive method for detecting the presence of pyrophosphate in TCP).

**Figure 4:**
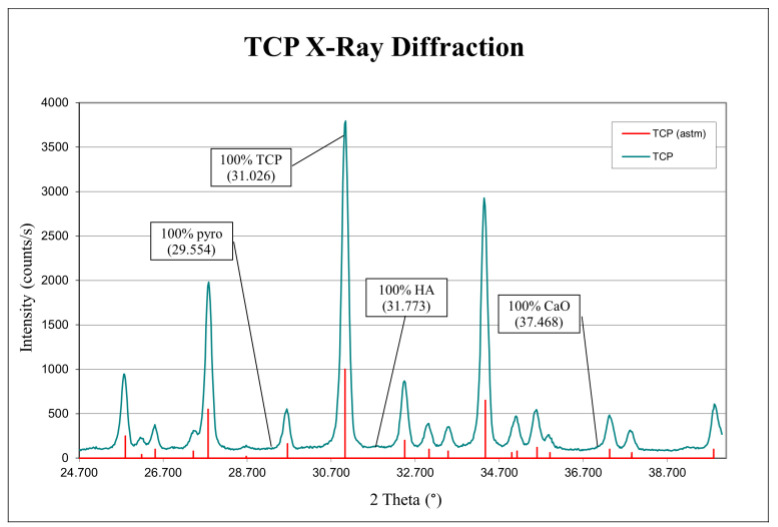
X-ray diffraction spectrum of a printed matrix. This illustrates the β-TCP and that no pyrophosphates, hydroxyapatite, or lime were detected.

**Figure 5:**
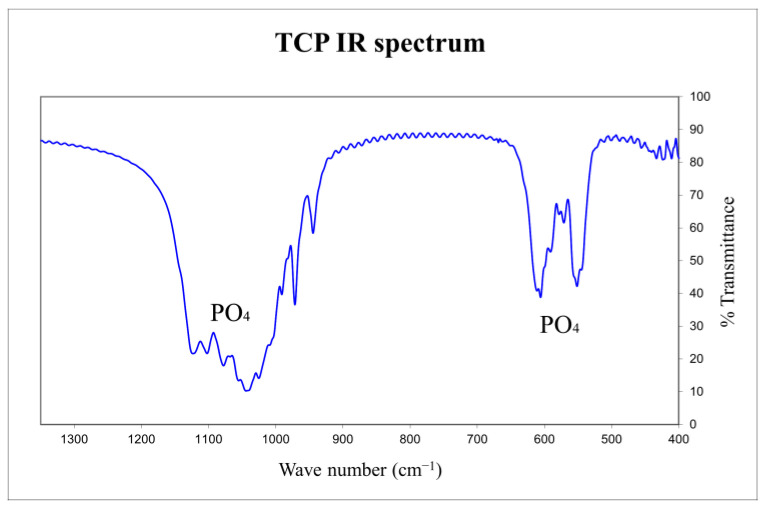
Infrared spectrum of the same printed matrix as in Figure 5, confirming that no pyrophosphate was detected.

Thus, we concluded that our powder production and our shaping techniques resulted in β-TCP matrices with negligible (possibly undetectable) traces (presence) of pyrophosphate and hydroxyapatite. Figure 4 and Figure 5 provide examples of the X-ray and infrared graphs that we obtained using a printed matrix.

### 3.2. Search for Pollutants or Trace Elements

The external research on pollutants in the powder is summarized in Table 1. They confirmed very few variations in material composition. All the tested high-risk contaminants were undetectable or showed very low levels, confirming the safety of our product for clinical use.

### 3.3. Evaluation of Open Porosity of Matrices

While taking these measurements, we observed and confirmed that the porosity of the matrices was linked to their sintering temperatures and not to the thickness of the print. A higher temperature leads to a decrease in open porosity. In fact, although we observed variability between samples, their porosity was about 20% for a sintering temperature of 1050 °C and decreased to 9 to 10% for a sintering temperature of 1120 °C. At the opposite end of the spectrum, despite the variations in printing thickness, the open porosity remained stable for a constant sintering temperature. Data are summarized in Table 2.

### 3.4. Evaluation of DLP Printing Accuracy

The results of our first test on cubes with 5 mm edges confirmed the accuracy of our printing technique while simultaneously demonstrating its limitations.

In fact, the dimensions of the edges were very close to what we expected. On the X and Y axes, the delta between the virtual model and printed pieces was a maximum of 0.03 mm. The Y-axis resolution seems to be less obviously connected to the printing technique, with a maximum deviation of 0.07 mm from our plan.

The second test confirmed these results, with a maximum deviation of 0.06 mm from our plan for the 5 mm cube and 0.07 mm for the 10 mm cube. These data are summarized in Table 3 and Table 4.

**Figure 6:**
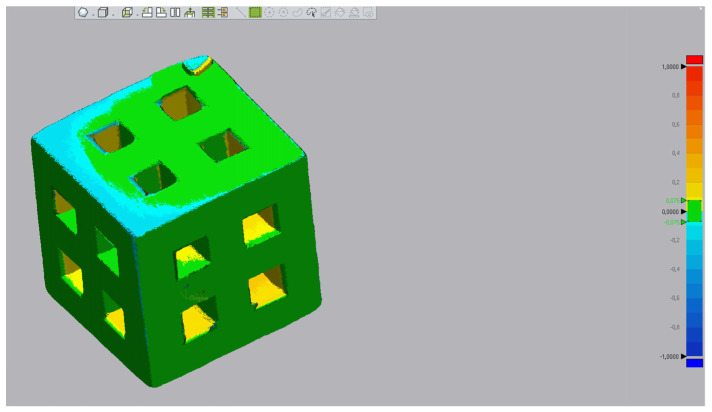
Comparison of 5 mm cube acquired by micro-CT scan with 3D design. Green represents the points with a tolerance under 75 microns.

At the opposite end, the study of window dimensions showed the limits of the DLP technique. In fact, we observed a maximum decrease in window size of 0.16 mm for the 5 mm cube (1 mm windows) and 0.21 mm for 10 mm cube (2 mm windows), both on the Z axis. These results are summarized in Table 5 and Table 6, and Figure 6 reports the visual evaluation of matrix deviation with a tolerance of 75 micrometres.

### 3.5. Release of Calcium Ions

We observed that the sintering temperature had a direct impact on the release of calcium ions. As expected, the increase in sintering temperature led to a drop in calcium release. For all samples, the maximum calcium release in the medium occurred within the first 12 h. This gradually decreased and stabilised without reaching a plateau phase.

After 6 days in pure water, considering the samples rinsed for 5 min, the maximum calcium release was reached with sample sintered at 1000 °C and was 10.67 mg/L, whereas it was 7.73 mg/L for the 1050 °C sample and only 6.3 mg for the 1100 °C sample.

It is also worth noting that rinsing the different samples reduced calcium release in the medium during the whole test. However, the maximum impact was observed with the lower sintering temperature and was reduced at 1050 °C and 1100 °C.

**Figure 7:**
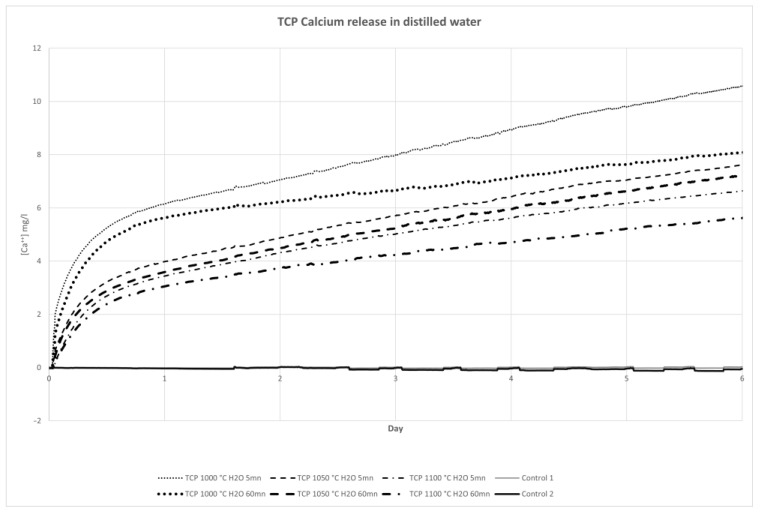
Calcium release in distilled water. Data are expressed in milligrams per litre.

Our data are summarized in Table 7 and Figure 7.

### 3.6. Safety of Printing Technique: Cell Proliferation

**Figure 8:**
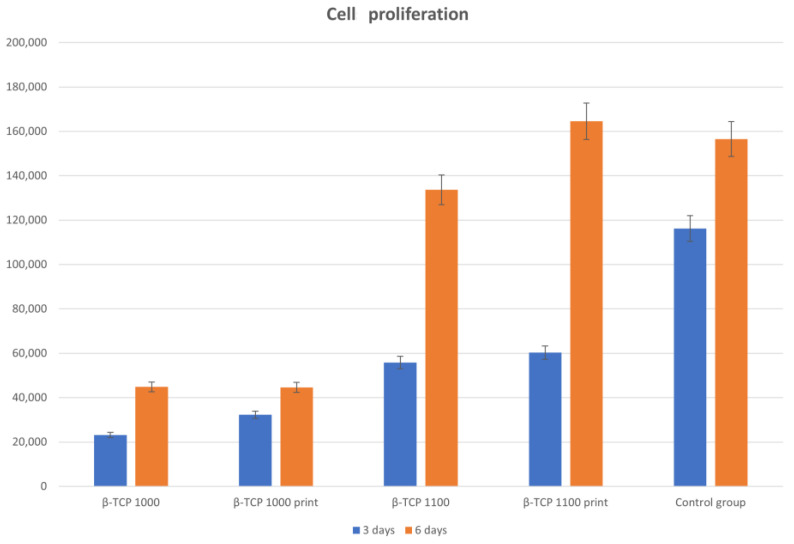
Comparison of cell proliferation after 3 and 6 days for in vitro cultures on cast and printed matrices at 1050 °C and 1100 °C.

The results of our first test on cells comparing casted and printed matrices for osteoblast cultures are summarized in Figure 8. We observed no significant differences between cultures on casted and printed matrices. The most influential factor was the sintering temperature, and we experienced an improvement in the proliferation of cells with higher sintering temperature. Finally, we also observed that osteoblasts improved their proliferation on matrices depending on the duration of culturing, with better results on matrices sintered at 1100 °C.

Our second test demonstrated that matrices that had undergone 1 h of rinsing before culturing exhibited increased osteoblast proliferation compared to our standard 5 min rinsing protocol.

**Figure 9:**
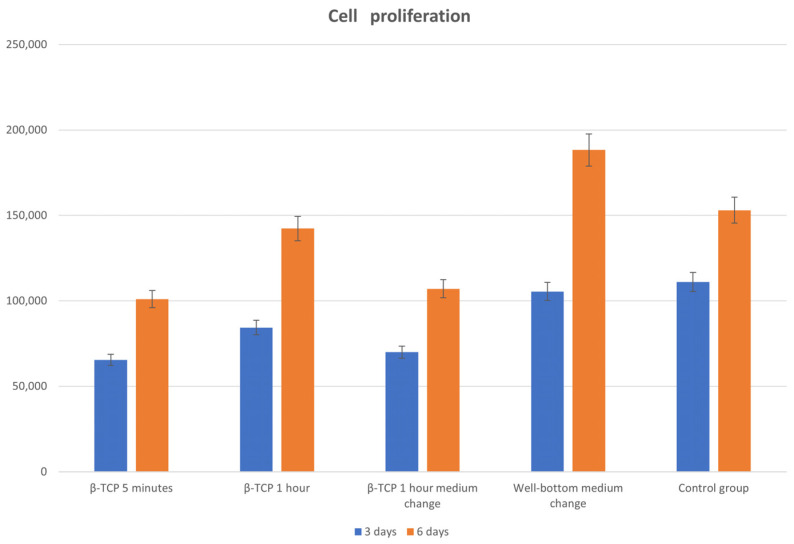
Evaluation of the effects of rinsing of matrices and changing of medium on cell proliferation on matrices sintered at 1050 °C.

Considering the daily replacement of culture medium, we observed that a precocious replacement seems to be prejudicial for cells; after a few days, however, fresh culture medium appears to be beneficial, as we can see in Figure 9.

## 4. Discussion

In our opinion, the data obtained in this study confirm that, as reported in the literature, the β-TCP matrices produced using our top-down DLP present promising properties for bone tissue engineering [33]. In fact, we demonstrated that the purity of β-TCP powder and the physico-chemical properties and biocompatibility of matrices are not altered by our printing process [34]. Our data also confirmed that DLP allows for reproducible and precise printing of phosphocalcic scaffolds. Our shaping technique therefore appears to be a solid basis for the development of phosphocalcic matrices with complex architectures incorporating different controlled macro-porosities and the microfluidic network needed to produce matrices for large-volume bone reconstruction.

### 4.1. β-TCP as a Bone Reconstruction Material

For decades, hydroxyapatite (HA) and β-TCP have demonstrated biocompatible and osteoconductive properties by allowing the attachment, migration, proliferation and differentiation of osteoblasts. Their osteoinductive properties, however, vary depending on their composition, geometry, specific surface area, and macro- and micro-porosity [35]. Taking full control of matrix production from powder synthesis to final shaping is fundamental for further biomedical applications. This observation led us to develop our own product.

β-TCP has a less stable crystal structure than HA and is more degraded by osteoclasts [36]. HA presents minimal resorption, whereas β-TCP can be completely resorbed into the reconstructed site and replaced by native bone, depending on its resorption speed. In our study, we confirmed that the matrices we produced possess resorption properties that vary according to the sintering temperature.

Although the resorption of β-TCP is beneficial for osteoinduction, it is detrimental for the homing of cells, bone ingrowth and, therefore, osteoconduction. There must therefore be a compromise between resorption and stability of the scaffolds [37].

Calcium phosphate synthesis is not the unique parameter necessary for understanding biological properties. As we observed, an increase in the sintering temperature leads to a decrease in the micro-porosity of the matrices, as well as their resorption. This is in agreement with the findings of previous research that reported a reduction in micro-porosity associated with an increase in the grain size and the specific surface area [38,39]. Our first in vitro culture confirmed the biological impact of the sintering temperature, resulting in better cell proliferation associated with a higher sintering temperature.

We also tested the rinsing of our matrices to eliminate the non-adherent particles resulting from the sintering step. We observed a decrease in calcium release for all sintering temperatures. These results are in keeping with our second in vitro cultures, which achieved better results on rinsed matrices.

Studying the results of both culture tests, we can conclude that higher sintering temperatures are always beneficial for osteoblast cultures. That said, it is very difficult to directly transfer our in vitro observations to in vivo results. In vitro, the stability of β-TCP matrices is advantageous, improving the homing properties of their cells. However, these results may greatly differ from in vivo cultures, which are much more complex systems with autoregulation processes in which the osteoinductive properties of β-TCP cannot be ignored. That is why we focused our study on matrices sintered at 1050 °C, constituting a good compromise for future in vivo studies.

### 4.2. Purity and Biocompatibility of 3D-Printed Matrices

In most cases, Fused Deposition Modelling (FDM) is used to 3D print biomedical scaffolds [40]. The FDM technique does, however, present a number of limitations in terms of resolution; for example, its large nozzles (around 0.4–0.5 mm) and speed of production mean that each point of the printed matrix must be individually extruded. The DLP 3D-printing system, which uses layer-by-layer polymerisation, has the potential to produce at better speeds, and, depending on the size of the printing surface, multiple matrices can be produced simultaneously. Moreover, the resolution of FDM is not sufficiently high [41]. DLP mixes phosphocalcic powders with polymer binders. Until recently, production techniques did not attempt to completely remove polymers to maintain the structural integrity of the matrices. Our study is, therefore, one of the first to report the production of 3D-printed pure phosphocalcic matrices [19,42,43]. In fact, our production process combines de-binding and sintering processes in a single step that allows for the destruction and removal of biological components present in matrices, including polymer binders.

Thus, X-ray diffraction and infrared spectroscopy confirmed the industrial quality of our production. Looking for pollutants confirmed that both our synthesis and printing processes are safe and did not introduce pollutants into the matrices. Study of the open micro-porosity also demonstrates that the proposed printing processes did not modify properties of the β-TCP. Open porosity is mandatory in completely removing polymers and providing easy revascularisation of the matrices. Finally, the results of our osteoblast culture did not significantly differ between printed and casted scaffolds, demonstrating that they have the same biological properties and confirming the biocompatibility of our printing technique.

In our opinion, these elements demonstrate the efficiency of our technique in producing biocompatible 3D-printed phosphocalcic matrices.

Even though our study focused on β-TCP, other calcium phosphate synthetic apatites, like HA and biphasic calcium phosphate (BCP), a mixture of hydroxyapatite and β-TCP, can also be considered because of their specific biological and mechanical properties [13,33]. However, depending on the synthetic calcium apatite composition, specific formulations of polymer binders are necessary if we want to obtain high-density matrices with optimised properties [44,45].

### 4.3. DLP Printing for Clinical Applications Related to Tissue Engineering

Rapid prototyping processes are of interest with regard to controlling the reproducible micro-porosity of matrices compared to conventional methods [23]. Indeed, conventional approaches result in variable and random interconnections due to the forming process. At the opposite end of the spectrum, as we experienced in our research, DLP enables us to produce reproducible matrices with controlled macro-porosity.

One of the difficulties involved in using DLP, however, finding a compromise between maximum powder loading and a viscosity compatible with the printing technique [46]. That is why in this article, we studied the accuracy of our printed devices with 68% solid loading, which is equivalent to conventional shaping and higher than most DLP printers.

In our opinion, our study confirmed the potential of our shaping process because of its simplicity and accuracy. One element to consider in our production technique is the sintering phase. This results in a homogeneous contraction of the green parts and, thus, improves the precision of part printing. The controls we tested by scanning different parts produced using the same 3D model showed marginal variations on the same axis. Determining the contraction of parts as a function of sintering temperature is therefore essential if we want to produce digital models of the parts to be printed. However, our data also expose the limits of our process, which are connected to the viscosity of our slurry, light scattering within the ceramic and printing speed. Those parameters are in keeping with the findings of the literature, and we can apply corrections in the future to improve these results [47]. Modulating the dimensions in the Z axis can therefore compensate for the deformation observed during our printing process. Furthermore, future modification of the light source during the printing process could also lead to an increase in our resolution, which we evaluated to be lower than 50 µm.

At present, most clinical practice involves the use of synthetic calcium phosphates in the form of powders or prefabricated blocks to fill bone defects. These, however, cannot perfectly adapt to reconstruct the defect, thus leading to instability and failure. Indeed, biomechanical stability is one of the fundamental elements involved during the bone healing process, and DLP printing of a custom implantable scaffold is of a high level of interest [48,49].

Furthermore, DLP allows us to design matrices with controlled macro-porosity that can be modified to mimic the characteristics of native bone, which has variable macro-porosity. The pores of the trabecular bone range in size from 200 to 700 µm and have a porosity ranging between 50 and 90%, whereas the cortical bone has less than 20% porosity and a pore range of 1100 µm. Furthermore, it has been demonstrated that variations in pore size seem to be essential to the biological properties of matrices [37]. A study using DLP to produce macro-porous BCP matrices compared the impact of the size of macro-pores ranging from 0.8 to 1.4 mm on bone formation. The authors concluded that the formation of new bone was satisfying but that no significant variation was observed after 8 weeks of culturing [19]. Other studies have reported that macro-pores with a diameter of up to 140 µm increase new bone formation and capillary density, that pores of up to 100 µm are important for bone oxygenation and that angiogenesis and pores of less than 1 µm play an important role in bioactivity [50,51].

In light of the above, the high-precision 3D printing of phosphocalcic bone matrices using techniques including our own is key to the development of bone tissue engineering. Such techniques allow for the precise control of the scaffolds’ architectures and optimise the physical and biological properties of scaffolds, enabling them to assume different shapes and have pores of a range of sizes.

Our study did not research the compressive strength of the matrices we produced. We arrived at this decision because the compressive strengths of phosphocalcic matrices increase with their sintering temperatures. In addition, the macro-porosity of the matrices and their various possible shapes have a direct impact on their mechanical strengths [19].

In tissue engineering, vascularisation is considered to be essential [52]. Most bone tissue engineering protocols use vascularisation brought by the surrounding tissues. Cells should be within 200 µm of a vessel in order to exchange oxygen and nutrients, thus theoretically limiting the size of functional bone scaffolds and preventing necrosis [53]. Large reconstructions also need to both mimic bone and incorporate the vascularisation or fluidic network to make them useful in clinical applications. Multiple bioprinting strategies have therefore been developed to this end, including extrusion-based bioprinting, inkjet printing, acoustic wave patterning and light-based bioprinting [54].

When it comes to phosphocalcic synthetic biomaterials, however, bioprinting is not compatible with the sintering process that is necessary for densification of the matrices. Another approach to mimic a functional vascularisation is to print a fluidic network into matrices and then culture endothelial cells on its walls. In that approach, the design of the network is essential to reduce the hypoxic areas inside matrix, as well as to contribute to a fluid flow with low turbulences, thus reducing thrombogenesis [55]. Given these various constraints, DLP printing appears promising for producing phosphocalcic matrices with both controlled macro-porosity and a “vascular tree”. And this is precisely why we developed our shaping technique.

At present, the printing of phosphocalcic matrices is one of the most promising fields in bone tissue engineering. And given the accuracy of DLP, it could become the gold standard. However, printing is only one of the steps that can affect the biological and mechanical properties of matrices.

Given the complexity and number of parameters to be considered in producing these scaffolds, the complete, careful monitoring of all steps in the production of these matrices—from powder synthesis to final sintering—is essential. This is reflected in our decision to dedicate a section of our study to the characterisation of our production to confirm that we produced β-TCP and that our printing technique does not alter its properties.

We also studied the accuracy of our shaping technique using DLP. Our findings demonstrate that top-down DLP is an effective technique for producing precise phosphocalcic matrices and that the shrinkage of green parts linked to sintering is involved in increasing the printing resolution. These findings will enable us to devote future studies to exploring complex phosphocalcic matrices, with a special focus on the development of a functional vascular network.

## Figures and Tables

**Table 1:** Search for pollutants or trace elements in the powder before and after the printing process (expressed in mg/kg).

Element	Before Printing	After Printing
Mercury/Hg	<0.050	<0.050
Arsenic/As	<1.0	<1.0
Lead/Pb	<1.0	<1.0
Cadmium/Cd	<1.0	<1.0
Stibium/Sb	<1.0	<1.0
Chromium/Cr	<1.0	<1.0
Nickel/Ni	<1.0	<1.0
Barium/Ba	2.7	2.8
Copper/Cu	<0.050	<1.0

All data before and after printing report that undetectable or to low levels of contaminants were present. A value of <1.0 means that the device could not detect a quantity less than 1 milligrams per kilogram, which is well below the acceptable limit values.

**Table 2:** Evaluation of open porosity of matrices.

Matrix Production Parameters	1	2	3	4	5	6	Mean
50 µm/1050 °C	18.40	22.44	19.80	21.92	21.57	21.32	20.91
100 µm/1050 °C	25.13	18.37	19.42	21.05	20.15	21.63	20.96
50 µm/1120 °C	10.10	7.68	12.51	11.32	9.03	8.97	9.94

Data are expressed in percentage of the open porosity of matrices.

**Table 3:** Evaluation of DLP accuracy using cubes edges (first test).

Edges	Cube A	Cube B
Average x	4.980	4.970
Standard deviation X	0.008	0.007
Average y	4.970	4.980
Standard deviation Y	0.019	0.008
Average z	4.930	4.960
Standard deviation Z	0.033	0.008

Average dimensions (X/Y/Z) are expressed in millimetres.

**Table 4:** Evaluation of DLP accuracy using cubes edges (second test).

Edges	5 mm Cube	10 mm Cube
Average x	4.980	9.980
Standard deviation X	0.004	0.010
Average y	4.980	9.980
Standard deviation Y	0.005	0.004
Average z	4.960	9.970
Standard deviation Z	0.009	0.032

Average dimensions (X/Y/Z) are expressed in millimetres.

**Table 5:** Evaluation of DLP accuracy using cube windows (first test).

Windows	Cube A	Cube B
Average x	0.890	0.890
Standard deviation X	0.008	0.010
Average y	0.900	0.890
Standard deviation Y	0.008	0.030
Average z	0.860	0.860
Standard deviation Z	0.009	0.015

Average dimensions (X/Y/Z) are expressed in millimetres.

**Table 6:** Evaluation of DLP accuracy using cubes windows (second test).

**Windows**	**5 mm Cube**	**10 mm Cube**
Average x	0.890	1.890
Standard deviation X	0.010	0.007
Average y	0.900	1.820
Standard deviation Y	0.017	0.022
Average z	0.870	1.790
Standard deviation Z	0.011	0.046

Average dimensions (X/Y/Z) are expressed in millimetres.

**Table 7:** Calcium release depending on sintering temperature and rinsing of matrices.

Matrix Parameters	3 h	6 h	12 h	1 Day	2 Days	3 Days	4 Days	5 Days	6 Days
1000 °C/5 min	2.50	3.60	5.35	6.24	7.15	8.86	9.01	9.89	10.67
1000 °C/60 min	2.40	3.50	4.84	5.72	6.31	6.75	7.23	7.74	8.19
1050 °C/5 min	0.95	2.20	3.01	4.09	4.97	5.80	6.52	7.15	7.73
1050 °C/60 min	0.94	2.20	2.97	3.70	4.60	5.35	6.05	6.72	7.32
1100 °C/5 min	0.90	1.60	2.52	3.60	4.47	5.14	5.70	6.28	6.30
1100 °C/60 min	0.86	1.56	2.48	3.19	3.85	4.35	4.80	5.30	5.73

Data are expressed in milligrams of calcium per litre.

## Data Availability

The authors confirm that the data can be obtained upon reasonable request to the corresponding author: thomaswojcik4@gmail.com.
